# Shape Memory Composite Sandwich Structures with Self-Healing Properties

**DOI:** 10.3390/polym13183056

**Published:** 2021-09-10

**Authors:** Fabrizio Quadrini, Denise Bellisario, Leandro Iorio, Loredana Santo, Panagiotis Pappas, Nikolaos Koutroumanis, George Anagnostopoulos, Costas Galiotis

**Affiliations:** 1 Department of Industrial Engineering, University of Rome ‘Tor Vergata’, Via del Politecnico 1, 00133 Rome, Italy; iorio@ing.uniroma2.it (L.I.); loredana.santo@uniroma2.it (L.S.); 2 Foundation for Research and Technology Hellas, Institute of Chemical Engineering and High Temperature Chemical Processes, University of Patras, Stadiou Str., Rio, GR 26504 Patras, Greece; ppappas@iceht.forth.gr (P.P.); nickkoutrou@iceht.forth.gr (N.K.); anagnostopoulos@iceht.forth.gr (G.A.); c.galiotis@iceht.forth.gr (C.G.); 3 Department of Chemical Engineering, University of Patras, University Campus, GR 26504 Patras, Greece

**Keywords:** self-healing capsule, shape memory properties, PU foams, micro-capsules, sandwich composite structures, shape memory polymer composites

## Abstract

In this study, Polyurea/Formaldehyde (PUF) microcapsules containing Dicyclopentadiene (DCPD) as a healing substance were fabricated in situ and mixed at relatively low concentrations (<2 wt%) with a thermosetting polyurethane (PU) foam used in turn as the core of a sandwich structure. The shape memory (SM) effect depended on the combination of the behavior of the PU foam core and the shape memory polymer composite (SMPC) laminate skins. SMPC laminates were manufactured by moulding commercial carbon fiber-reinforced (CFR) prepregs with a SM polymer interlayer. At first, PU foam samples, with and without microcapsules, were mechanically tested. After, PU foam was inserted into the SMPC sandwich structure. Damage tests were carried out by compression and bending to deform and break the PU foam cells, and then assess the structure self-healing (SH) and recovery capabilities. Both SM and SH responses were rapid and thermally activated (120 °C). The CFR-SMPC skins and the PU foam core enable the sandwich to exhibit excellent SM properties with a shape recovery ratio up to 99% (initial configuration recovery). Moreover, the integration of microcapsules (0.5 wt%) enables SH functionality with a structural restoration up to 98%. This simple process makes this sandwich structure ideal for different industrial applications.

## 1. Introduction

In recent decades, shape memory (SM) materials and structures have aroused increasing interest related to new usage opportunities and opened up different fields of application. In particular, SM polymers and composites can recover their permanent shape from a temporary shape under suitable external stimuli [1,2,3,4,5,6]. Specifically, SM polymers (SMPs) have been applied in various fields such as biomedical devices, deployable structures and actuators [7,8]. The distinctive aspect of shape recovery is the possibility of returning spatially to the original shape. This could aim and contribute to the external shape recovery of a structure, for example, after a damage. However, there is not a real repair without reactivating and re-establishing the material continuity. In fact, the self-healing effect refers to the ability of the material to be repaired automatically or by external stimuli after being damaged or cracked [9,10]. In this light, the addition of self-healing functionality through the addition of suitable SH materials is essential to complete the repairing action. The SH performance is mainly achieved in two ways: with either intrinsic or extrinsic methods. The former methods allow the micro-cracks to heal without using healing agents, but with reversible reactions (non-covalent or dynamic covalent bonds) [11,12,13]. Conversely, the latter, extrinsic methods are based on healing agents embedded in microcapsules, or vascular networks, and their action is related to the release of the agents that therefore heal the micro-cracks [14]. This second method grants the possibility to add SH properties to commercial polymers since no adaptation of matrix molecules is necessary [15]. The combination and integration of these two smart performances, shape memory and self-healing, has been extensively studied in recent years, especially for polymers, composites and coatings [16,17,18,19], in order to improve the reliability, extend the service lifetime and reduce maintenance costs [20,21]. As temperature is a central stimulus for SH effect, and the healing temperature may differ significantly, it should be compatible with the external stimulus for the shape recovery.

Different methods have been used to combine SM properties with SH performances, such as relating SMP fibers and microcapsules, harboring a healing agent, preparing simple solution blending or incorporating fusible healing agents in host matrices [19,22,23,24]. For example, Kong et al. fabricated a self-healing high-temperature shape memory polymer by integrating fusible thermoplastic polystyrene (PS) into a shape memory polyimide matrix, which exhibits very good shape memory effects [25]. Jony et al. studied macro fiber composite (MFC)-assisted in situ healing of a unidirectional fiber-reinforced polymer (FRP) composite, in which the healing of damage in carbon fiber-reinforced composite (CFRP) was achieved by using a blend of biphasic healant: polycaprolactone (PCL) and polyurethane shape memory polymer (SMP) [26].

In comparison, a combination of SM and SH performances in sandwich structures has been scarcely studied [27]. Although these structures are widely used in many engineering applications, such as space shuttles, aircrafts, ships, cars, wind-turbine blades or buildings, they are subjected to critical problems such as transverse load-induced impact damages. In particular, in composite sandwich structure, the skins are responsible for protecting and shielding the core as well as supporting bending loads, while the core is able to separate and fix the skins, to resist transverse shear and in-plane load, and entering other functions such as absorbing impacts or insulating heat transfer. The ability of the core to host other functionalities made it possible to evaluate the possibility to add self-healing properties into the sandwich core. In this regard Williams et al. designed and tested vascular self-healing systems for foam-cored sandwich panels, which, after damage and self-healing, show a strength comparable to the undamaged ones [28]. Moreover, the core of a composite sandwich structure is very often a polymer foam, and recently SMP foams with integrated self-healing properties were proposed. In particular, Li and Xu and Li et al. suggested a SMP syntactic foam sealant for a compression-sealed expansion joint in a bridge floor or concrete roadway [29,30]. The integration of these SMP-based syntactic foams as cores of sandwich structures was then formulated by Behl et al., who proposed the external confinement of fiber-reinforced polymer skins as an out-of-plane constraint to the SMP core [31]. In detail, Behl et al. fabricated the syntactic foam by dispersing glass hollow spheres and multi-walled carbon nanotubes into a SM polystyrene matrix without the direct use of particles for extrinsic self-healing. Thus, they studied the shape memory effect alone with the partial confinement of the sandwich skin and demonstrated the crack-closing capability by the SM effect of the SMP core. However, a combination of SM with self-healing systems based on healing agents can be interesting and almost crucial for such composite sandwich structures. However, a combination of SM with self-healing systems based on healing agents can be interesting and almost crucial for such composite sandwich structures. Previous conclusions in this regard were made by authors with shape memory composite sandwiches with self-healing properties for marine applications [32]. In particular, this first approach was made to simulate the self-healing function through the fabrication of sandwiches with thin carbon fiber laminates for skins and SMP foams for core. In that case, once ruptured, the failure zone was covered with uncured epoxy resin in liquid state and after was recovered. However, the study was limited to a post-rupture action that simulated the insertion of a healing agent.

For this reason, the current study introduces design, manufacturing, as well as the self-healing ability of a shape memory composite sandwich structure composed of two SMPC laminates as skins and a SM polyurethane foam with embedded PUF/DCPD microcapsules as core. The synthesis of the SH capsules followed a well-established method [33] which was modified mainly aiming to achieve an increased efficiency and lower fluctuations in sizes. The fabrication of the capsules was followed by storage at deep freeze conditions (−18 °C) until the incorporation into PU foam matrices. In order to evaluate shape memory and self-healing performances, the PUF/DCPD microcapsules underwent severe compression and thermal recovery tests, first separately, and then inside the PU foams through micrographs. Subsequently, the PU foams, with and without microcapsules, have been used as the core of composite sandwiches whose skins were fabricated using two carbon fiber prepreg layers with an SM epoxy interlayer. This SMPC was already studied by authors for aerospace application in other works [34,35] and exhibits very good SM properties. The shape memory and self-healing performances of the fabricated sandwich structures were further, and systematically, investigated by bending and thermal recovery tests.

## 2. Materials and Methods

### 2.1. Materials

#### 2.1.1. Self-Healing Capsules Fabrication

Self-healing (PUF/DCPD) microcapsules were fabricated using a modified recipe based on that of Brown et al. [33] The main differences can be allocated to the implementation of a tip sonication step after the addition of formaldehyde, a short-range agitation process and an alternative humidity removal step using inert gas flow.

**Figure 1:**
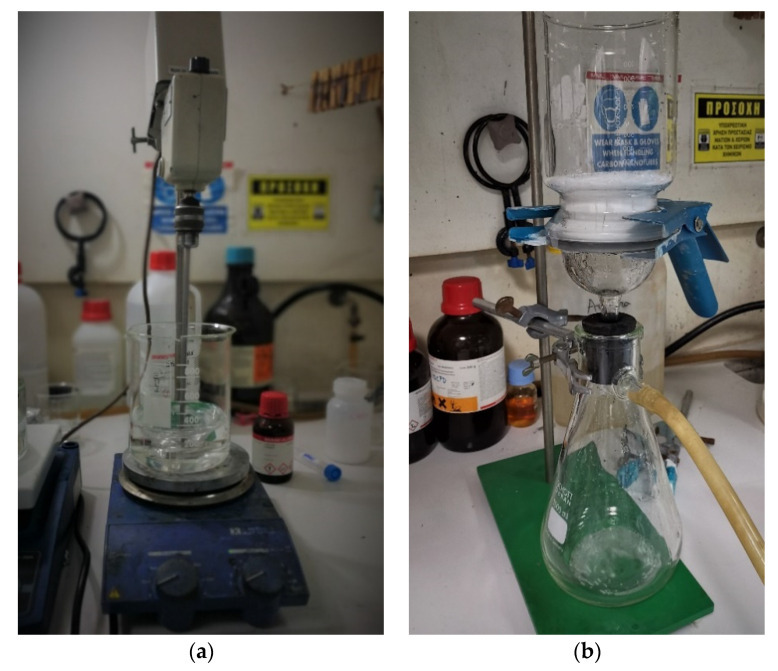
Snapshots from the SH capsules fabrication process: (**a**) sonicator use; (**b**) vacuum filtering and drying.

The procedure is initiated by mixing ethyl methyl acrylate (EMA) copolymer (~25 mL), by Sigma Aldich^®^, Steinheim, D-89555, Germany, with deionized water (100 mL) under agitation at room temperature. The outer shell wall ingredients are added subsequently in the mixture: 2.5 g urea, 0.25 g ammonium chloride and 0.25 g resorcinol, all by Sigma Aldich^®^, Steinheim, D-89555, Germany. The solution is then transferred to a temperature-controlled water bath and the pH is adjusted to 3.5 by careful step-wise addition of NaOH, by Honeywell, Seelze, D-30926, Germany. Using a blade propeller, by Heidolph Instruments^®^, Schwabach, D-91126, Germany, the aqueous solution is mechanically agitated at 500 rpm; while aiming to minimize the formation of bubbles, 1-octanol is added, by Sigma Aldich^®^, Steinheim, D-89555, Germany. The next step involves the addition of 30 mL of DCPD, by Sigma Aldich^®^, Steinheim, D-89555, Germany, upon the formation of a fine droplet suspension. The stabilization of the solution (~15 min) is followed by addition of formaldehyde (formalin), by Honeywell, Seelze, D-30926, Germany, in aqueous dispersion (37% wt, 6.35 g), increase in the agitation speed at 800 rpm and utilization of a 10 mm tip sonicator (Sonotrode LS24d10), by Hielscher Ultrasonics^®^, Teltow, 14513, Germany, for 5 min (Figure 1). During the process, the temperature rises from RT to 55 °C at a rate of 2 °C/min, and the emulsion is protected against evaporation with an aluminum foil. After 4 h, the mixture is allowed to cool down to RT, followed by vacuum filtering and drying in open air for 2 h. Finally, it is placed in a cabinet overnight, with continuous nitrogen flow.

The storage of the produced capsules is carried out under deep freeze conditions in order to avoid any deterioration due to temperature or UV radiation. As reported by Brown et al., the core DCPD monomer melts at 38 °C, while the remaining moisture and formaldehyde are eliminated at ~105 °C [33]. The shell of the capsule is reported to decompose at the range of 225 to 258 °C [36].

#### 2.1.2. Shape Memory Composite Laminates

The shape memory polymer composite (SMPC) laminates were fabricated using commercially available materials. The SMPC was manufactured with two external layers of carbon fiber prepreg and an interlayer of shape memory (SM) resin. In particular, the prepreg used was an HexPly^®^ M49/42%/200P/CHS-3K by Hexel^®^, Stamford, CT 06901, USA. This prepreg had a thermosetting matrix (epoxy resin HexPly^®^M49 with a glass transition temperature of 120 °C) reinforced with carbon fiber fabric, which had a 1 × 1 plain structure. The nominal properties of the fabric are a thickness of 0.3 mm, fiber density of 1.78 g/cm^3^, and resin content of 42 wt%. The SM interlayer consists of an uncured epoxy resin (3M Scotchkote 206 N by 3M, Hudson Rd, Maplewood, MN 2501, United States,) in the form of green powder. This is a heat curable thermosetting epoxy coating with a density of 1.44 g/cm3. During SMPC manufacturing, an interlayer of 100 μm thick SM resin powder was uniformly deposited between two prepreg layers before molding operations. The detailed fabrication procedure of SMPC laminates and their shape memory performances were already published by authors [34,35]. In particular, two laminates for each sandwich structure were fabricated, and these laminates were a two-ply SMPC with a shape memory recovery ratio of about 97 ± 3% [37].

In terms of durability, the shape memory efficiency in multiple cyclic tests of SMPC skins has been already evaluated in previous works [38]. Results show that negligible effects are present for the SMPC laminate of the skin at least for limited number of cycles if the elastic range is not crossed during the memory steps. In the case of foams, a training effect may be also observed at the first cycle [39].

#### 2.1.3. Polyurethane Foams

The polyurethane (PU) foams were fabricated by mixing the two polyurethane thermosetting components of ESPAK 90 supplied by Prochima, 61036—Colli al Metauro (PU), Italy. The reagents are freon-free and their mix at room temperature generates a rigid closed-cell microstructure with a nominal density of 90 kg/m^3^ and a volume increase of 12 times in free foaming conditions. The two components are named A and B and their nominal volume mixing ratio is 100/100. Table 1 summarizes the physical properties of the ESPAK90 components.

The used polyurethane resin is rigid at room temperature. Its glass transition temperature, after curing, has been measured to be 70 °C. Therefore, its elastic recovery at room temperature is very low. For the definition of the recovery temperature in the test, it was important this temperature was high enough to allow polyurethane resin and SMPC shape recovery as well as self-healing agent activation. Excess of temperature must be avoided to reduce the effect of organic material degradation.

**Figure 2:**
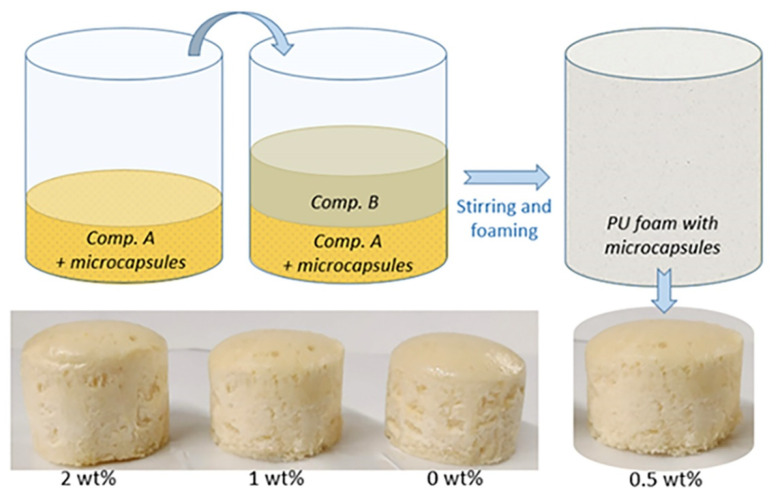
Scheme of the foaming process with embedding of microcapsules and fabricated PU cylindrical foams.

In order to achieve greater repeatability of the foaming conditions, the mixing ratio was based on the weighting of the components instead of using volume nominal density values. Under these imposed mass conditions for reagents, the resulting foaming ratio was equal to 8 considering the same foaming volume. This mixing condition also allows us to insert the micro-capsules as a percentage by weight of the reagents. In particular, for the fabrication of foams with embedded micro-capsules, the PUF/DCPD capsules were added to the more viscous component A and afterward mixed with B to start the foaming process (Figure 2).

This procedure enables us to uniformly distribute the microcapsules within the foam. Microcapsules have been added to PU reagents in increasing percentages of 0, 0.5, 1 and 2 wt% in order to choose and evaluate the better percentage for the self-healing effect. Several PU foam samples with microcapsules were fabricated using a cylinder with a volume of 20.817 cm^3^ (20 mm of diameter) as foaming mold. After foaming, the samples were cut at the base and at the top to get a more regular shape.

#### 2.1.4. SM Sandwich Structures

**Figure 3:**
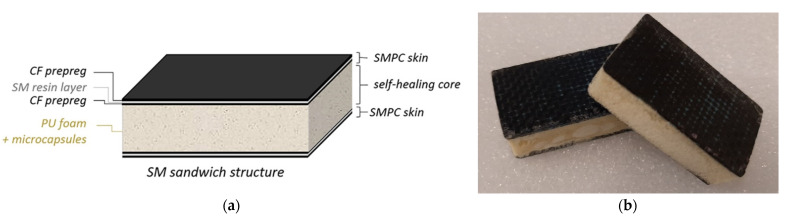
(**a**) SM sandwich structure; (**b**) image of the manufactured sandwich samples.

The shape memory (SM) sandwich structures were fabricated using two SMPC samples of 100 × 30 mm^2^ as skins and the PU foam with and without microcapsules as the core of the structure (Figure 3a). The PU foam was foamed directly between the SMPC skins inside a mold in order to fill a total volume of 50 × 30 × 12 mm^3^. Such foaming volume allows it to reach a foaming ratio of about 4.5. A schematization of the sandwich structure is shown in Figure 3a. Two samples for each condition, with and without microcapsules in the foam’s core, were fabricated (Figure 3b). In particular, a weight percentage of 0.5 was chosen for the samples with microcapsules.

### 2.2. Methods of Experimental Evaluation

#### 2.2.1. SH Capsule Characterization by Means of Optical Microscopy

**Figure 4:**
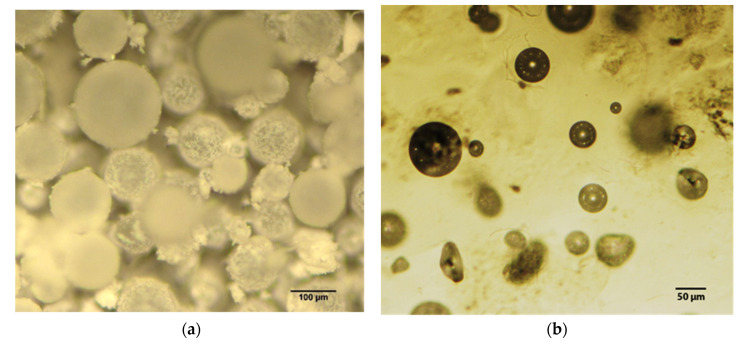
(**a**) Post-production optical microscopy image of SH capsules; (**b**) image SH capsules embedded in water-clear epoxy resin.

Optical microscopy study of the produced SH capsules batch was carried out in order to verify the efficiency of the process and calculate the mean size of the capsules (Figure 4). Moreover, a small quantity of capsules was embedded into water-clear epoxy matrix, cured at RT, aiming to obtain more resolved and isolated images for better measurements. The mean size of the capsules was calculated as equal to 80 μm.

#### 2.2.2. PU Foams Evaluation

The density of the cylindrical foams was measured in order to evaluate the influence of microcapsule embedding in the foaming process. The sizes and the weights of the cylindrical foams were evaluated by a caliper with a sensitivity of 0.01 mm and an electronic balance with a sensitivity of 0.001 g, respectively.

Polymeric foams typically show an intrinsic shape memory effect and, in particular, some polyurethane foams demonstrate shape memory performances [39,40,41,42]. Thus, in order to evaluate the shape memory properties of this type of commercial polyurethane, extra shape memory measurements were performed in addition to the healing ones. Accordingly, the shape memory and self-healing tests on cylindrical PU foams were performed by means of two compression tests on a universal testing machine (MTS Insight 5) on virgin foams and after thermal healing. In particular, cold compression tests were carried out on foam samples at room temperature, with a displacement speed of 2 mm/min, a maximum vertical strain of 0.5 and maintenance time in the final displacement of 5 min. As a result, a severe deformation in the foam shape and the collapse and breakage of part of the foam cells and of microcapsules is achieved. Afterwards, the compressed samples were heated by a hot air gun for the recovery phase. The chosen temperature and time for the recovery phase were 120 °C and 5 min, respectively, thus achieving the double effect of shape recovery and self-healing consolidation. Subsequently, the recovered samples were tested again by compression with the same testing parameters. This cyclical procedure enabled the evaluation of both shape memory effect and self-healing performances. The shape memory effect was quantified by means of the shape fixity Sf and shape recovery Sr:

S_f_ = ε_u_/ε_f_ ∙100; (1)

S_r_ = ε_r_/ε_f_ ∙100; (2)

In particular, cylindrical foams were compressed at a fixed strain (ε_f_), then the unloaded temporary shape was evaluated (ε_u_), and after heat exposure, the recovered shape was measured (ε_r_). In this case, neglecting the slight variations in diameter, the heights of the foams were measured, in their initial state, after unloading and after recovery. Conversely, the self-healing performance was determined by a self-healing efficiency calculation [14]. The healing efficiency (η_1_) was calculated in relation to the compression stress at a fixed strain value:

η_1_ = σ_healed_/σ_initial_ ∙100; (3)

where σ_initial_ is the achieved stress at a fixed strain before damage and σ_healed_ is the stress achieved at the same strain value after the specimen has recovered. Moreover, the healing efficiency in relation to stiffness during loading (η2) was calculated as:

η_2_ = R_healed_/R_initial_ ∙100; (4)

where R_healed_ and R_initial_ are the stiffness of a specimen before damage and after recovery, respectively.

The PU foam structures before and after healing were observed with an optical microscope, Nikon Eclipse 80i, in order to investigate the microstructure of healed foam and interaction between closed cell-cracks/microcapsules.

#### 2.2.3. SM Sandwich Structures Evaluation

**Figure 5:**
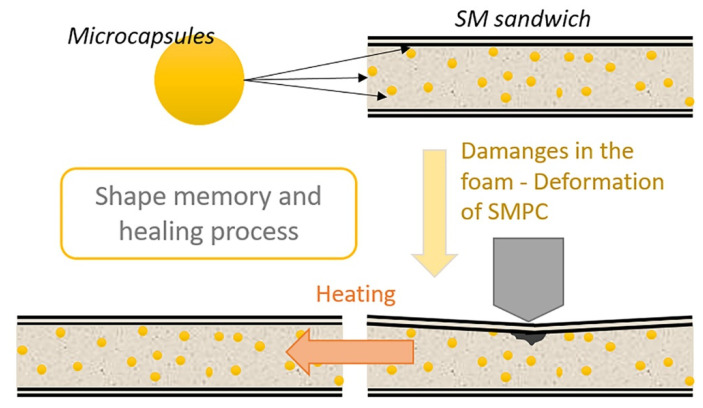
The shape memory and healing process schematization.

The self-repairing capabilities of the fabricated SM sandwich structures were evaluated via a three-point bending test using a universal testing machine (MTS Insight 5) as shown in the schematization in Figure 5.

The three-point bending parameters were: 10 N as preload, 40 mm as span-length and 1 mm/min as rate. The maximum vertical displacement was fixed at 6.5 mm, which was the maximum possible inflection for the sample without touching the supports of the test equipment. Moreover, this displacement allows us to introduce some cracks into the foam’s core without compromising the SMPC skins. The maintenance time in the maximum inflection configuration was set at 5 min, so as to fix the temporary shape. Afterwards the sample was unloaded and recovered through a heating step with a hot air gun (≈120 °C) for 10 min. After recovery, the sandwich sample has been tested again in the same bending configuration. The shape memory effect was evaluated by a bending test using Equations (1) and (2). The maximum inflection at a fixed bending angle (θf), the fixity angle after the release of the bending stress (θu) and the recovered bending angle (θr) after heating were evaluated by using a Sony Cybershot camera with an image software which allowed us to evaluate the bending angles. Similarly, to what was carried out for cylindrical PU foams, the self-healing effect was demonstrated by calculating the healing efficiencies (η_1_ and η_2_) using Equations (3) and (4).

## 3. Results

### 3.1. Sample Structures

The PU foams with and without micro-capsules were measured, and the values reported in Table 2. The foam heights shown in Table 2 are the maximum foaming heights. Density and compression tests were performed on cylindrical samples of the foam with flat and parallel faces.

**Figure 6:**
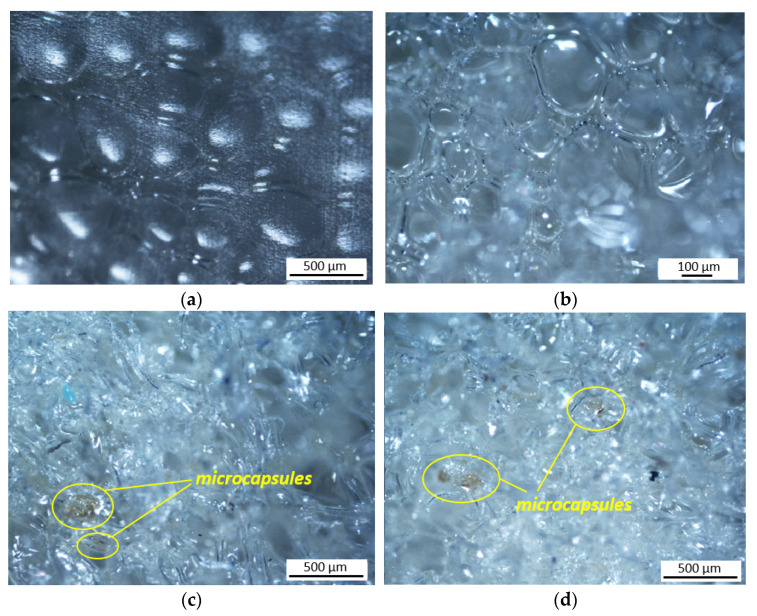
(**a**,**b**) optical microscopy images of PU foam without microcapsules; (**c**,**d**) images of SH capsules within the PU foam.

Micrographs of the PU foams with and without microcapsules are shown in Figure 6. Given the three-dimensional nature of the foam, in order to avoid cell breakage and prevent the observability of the microcapsules, the foams were observed from the outer surfaces. The PU foam exhibits a closed cells structure wherein microcapsules of a darker color with sizes of several micrometers are distributed, as shown in Figure 6c,d.

### 3.2. Mechanical Properties, Shape Memory Effect and Self-Healing Performance of PU Foams

To understand the behavior of the self-healable PU foams under compression loading, the compression tests were performed before and after the recovery phase. Cold compression tests were carried out on foam samples at room temperature at the rate of 2 mm/min up to the maximum strain of 0.5. The maximum load was hold for 5 min. Test samples were cylindrical with a nominal size of 35 mm in diameter and 30 mm in height; real sizes are reported in Table 2 together with physical data.

**Figure 7:**
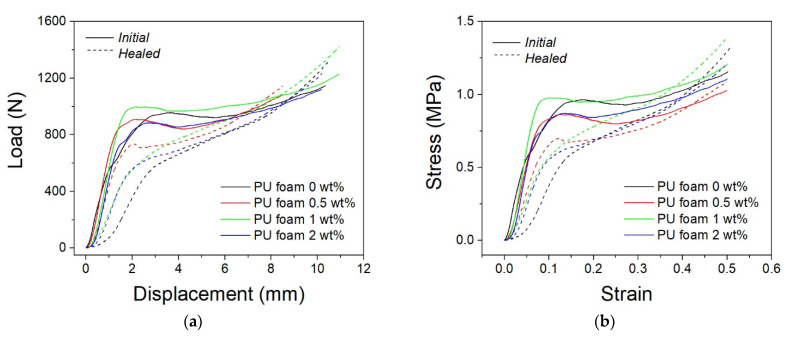
Load–displacement (**a**) and stress–strain (**b**) curves of PU cylindrical foams at initial state and after recovery.

Important boundary effects are expected during foam testing because of the complex interaction of the porous constructs with the compression platens and possible errors of parallelism between the contact surfaces. For this reason, stiffness data from the compression curves of foams were extracted together with stress data at fixed strains, and tests were repeated on two different samples. The compressive curves of initial and healed PU foams, with and without embedded microcapsules, are shown in Figure 7. The initial PU foams show stress–strain curves characteristic of elastic cellular solids with three zones. The first one is the initial rise connected to the elastic flexion of the cell walls; then there is the plateau related to the buckling of the walls, and it ends with a sudden increase linked to the densification of the cells with the approach of the walls to each other [43].

The shape of the compression curves for different filling levels is different before and after the healing step. The nominal behavior is given by the unfilled foam where a relative load peak is present between the initial pseudo-elastic linear range and the following small plateau region. The plateau depends on the cell collapsing and ends with a load increase due to foam densification, as already mentioned. Self-healing particles change this nominal behavior for the combination of several mechanisms: morphological changes of the foam cells, foam stiffness alteration, possible partial activation of the healing effect. As these mechanisms may have different efficiency on the basis of the amount of filling, the final curve shape is difficult to be predicted. This difference is highlighted by the healing effect when resin crosslinking occurs. Additionally, in this case, the nominal behavior is given by the unfilled foam where the plateau disappears, as well as a stable initial pseudo-elastic linear range. Only the densification part of the curve is repeated. In the presence of healing agents, different shapes are observable. These curves provide the first evidence that the presence of the healing agent is not always positive as some negative mechanisms in the initial compression curve may be compensated by some positive effects during self-healing.

In particular, it was observed that initial neat PU foam exhibits a compressive strength at 7% of strain of 0.66 MPa, while PU foam samples with 0.5, 1 and 2 wt% of microcapsules manifest values of 0.73, 0.88 and 0.71 MPa, respectively. This slight increase in the compressive strength is also related to an increase in the foam stiffness due to the microcapsule embedding. In particular, this is more evident in the sample with 1 wt% of microcapsules for which the calculated stiffness is 774.5 N/mm, while it was 631.4 and 671.4 for the 0.5 wt% and 2 wt% samples, respectively. This means that by adding the microcapsules, the compression stiffness was firstly increased (up to 1 wt%), and after decreased. By increasing the percentage of microcapsules from 0.5 to 1 wt%, the compressive stiffness was increased by approximately 20%. However, by comparing the PU foam containing the 2 wt% microcapsules and the pristine foam, the stiffness was also increased by about 7%. After compression, the damaged PU foam samples were heated and subsequently compressed again with the same testing conditions.

**Figure 8:**
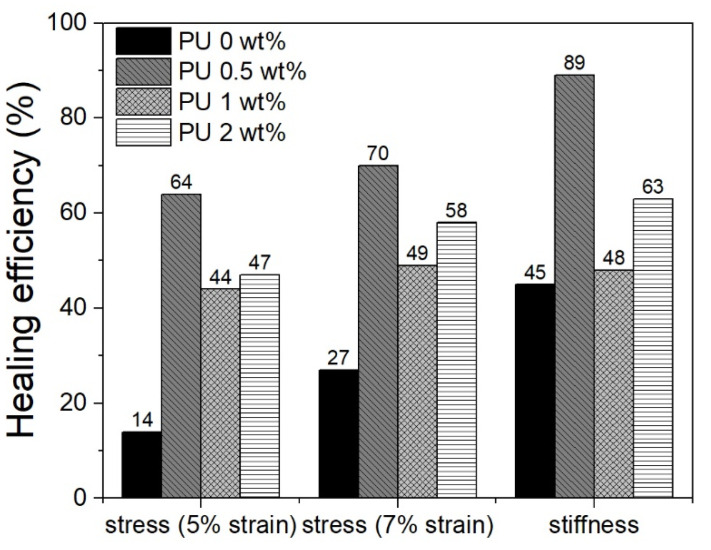
Healing performances of PU cylindrical foams after recovery.

**Figure 9:**
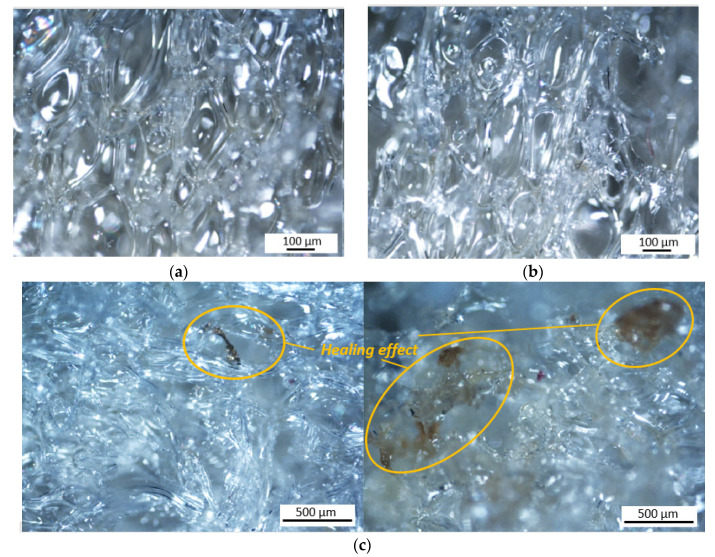
(**a**,**b**) optical microscopy images of PU foam after compression (shape fixity) before recovery; (**c**) images of PU foam after recovery (healing effect).

The curves after the healing phase are shown dashed in Figure 7. Healing efficiency is defined as the ratio of compressive stress at a fixed strain value (5% and 7%) after self-healing to that of the initial specimen. The obtained results are illustrated in Figure 8, together with healing efficiency assessed on the basis of stiffness values before and after healing. The healing effect was also observed through micrographs of damaged areas both in pre-healing (compressed sample) and post-healing (after heating), as shown in Figure 9. Micrographs make it possible to distinguish the presence and breakage of the microcapsules and the repair action of broken cells. PUF/DCPD plays the role of a healing agent, and the healing efficiency seems to have a great amount of around 0.5 wt% of microcapsule content.

**Figure 10:**
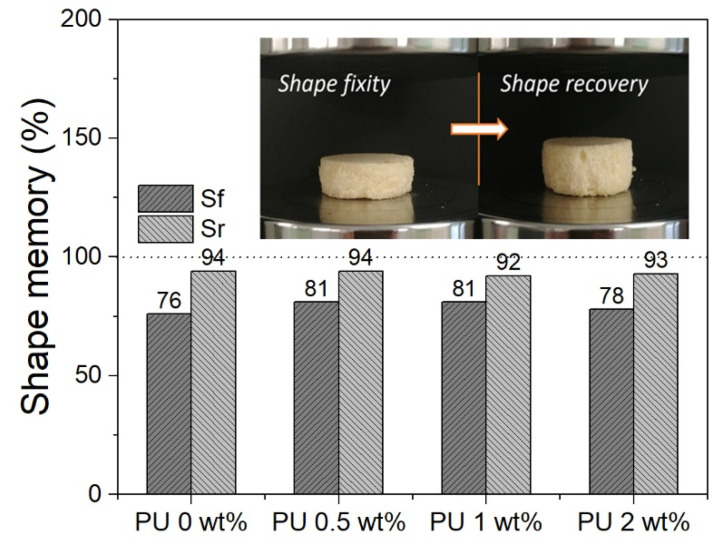
Shape memory performances of PU cylindrical foams with and without microcapsules.

Moreover, before and after compression tests, the shape memory effect of the foams was also evaluated. The measured values of shape fixity and shape recovery are shown in Figure 10. Regardless of the presence of microcapsules, the results show that the PU foam has a shape recovery ability of about 94% and a fixity one of about 79%.

### 3.3. Mechanical Properties, Shape Memory Effect and Self-Healing Performance of SM Sandwiches

**Figure 11:**
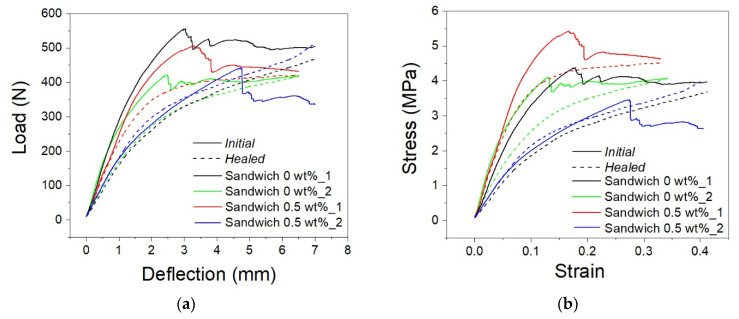
Load–displacement (**a**) and stress–strain (**b**) curves of SM sandwiches at initial state and after recovery.

Based on the results obtained for the PU foams, the shape memory sandwich panels were manufactured using the PU foam with 0.5 wt% of microcapsules as the core. The SM sandwich panels were tested with a three-point bending configuration up to a maximum strain of 40%. Figure 11a,b show the load–deflection and stress–strain curves for the SM sandwiches with and without microcapsules in the foam core at the initial state and after healing. Strain data from compression and bending curves were extracted from the measured crosshead of the testing machine by normalizing with real sample sizes.

**Figure 12:**
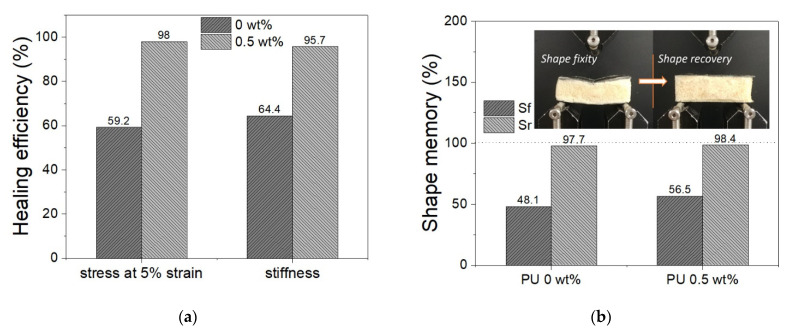
Load–displacement (**a**) and stress–strain (**b**) curves of SM sandwiches at initial state and after recovery.

The curves show an initial sudden rise in the stress up to a maximum, after which there are sharp variations related to foam breakage and to small de-bonding phenomena among SMPC skins and cores at the ends of the sample. In general, the maximum stress values and the stiffnesses detectable for samples with and without microcapsules are variable due to the inherent variability in the manufacturing process. In fact, the presented manufacturing process of both foam and composite skins is purely manual and suffers defects that can lead to less repeatability, as underlined by the differences between the experiments. In particular, because of the very large difference in stiffness between the foam core and the composite skins, small discrepancies in the thickness of the skins result in a large change in the sandwich performances. For this reason, results are discussed by considering the relative changes of each single specimen before and after the healing step. In particular, for the same tested sample it is possible to observe the trend before (initial) and after the thermal heating process (healed). Specifically, after the initial bending, the SM sandwich panels have been deformed and damaged. The heating step allows the recovery and healing of the panel system. After heating and cooling, the sample was tested again. It is evident that within the same experiment the initial curve is superimposable after healing with 0.5% of microcapsules. This does not happen in the absence of microcapsules: the initial slope of those curves is not repeated by tests after heating. The heating phase allowed the partial recovery of both shape and properties. Regardless, in order to be able to evaluate the healing performance, the healing efficiencies were calculated on the basis of the flexural stiffness and of the stress value at the fixed strain of 5%. Healing efficiency values are shown in Figure 12a. The samples with 0.5 wt% microcapsules in the core show an increase in healing efficiency of 31% for stiffness and of 39% for stress at 5% of strain. Then, analyzing the shape memory effect of the sandwich panels, in Figure 12b it is observable how shape fixity and shape recovery are quite the same for all the samples, regardless of the presence of microcapsules. The restoration of SM composite skin alignment and the foam elastic support affect the shape fixity that turns out to be around 50%. On the other hand, the shape recovery is promoted by the same factors and reaches values close to 98.5%.

## 4. Discussion

A first analysis of the structure of the fabricated foams with microcapsules allowed us to observe how the foaming heights seem to be minimally affected by the percentage of microcapsules added. In particular, there is a slight correlation in the height of the foams as the content of microcapsules increases. This can be partly linked to a greater presence of microcapsules which can be broken during mixing and foaming phases. On the other hand, given the same polyurethane nature of the microcapsules content and their low adding percentage, the density was not affected by the content of microcapsules or by the possible healing agent that flowed during embedding.

Analyzing the healing behavior of the PU foams with and without microcapsules in the absence of SMPC skins is possible to underline how a small addition of microcapsules greatly aids the self-healing capabilities of the cellular structure. In particular, regardless of the type of parameter used to assess healing efficiency, samples with 0.5 wt% microcapsules show higher efficiency levels. The foam sample with 0.5 wt% of microcapsules shows an increase in the healing efficiency between 44 and 50% compared to the pristine sample (0 wt%). For higher percentages of added microcapsules, there is a slight deterioration in healing performance with an increase of only 10–35% compared to pristine foam. In particular, the sample with 1 wt% of microcapsules shows the worst healing performance. This can be linked to a greater number of broken capsules during the preliminary mixing and foaming phases, as also evidenced by the initial foaming heights. In addition, as already noted, the foams have intrinsic shape memory properties that in this case assist the micro-healing effect of the capsules. In fact, regardless of the presence of microcapsules, there is an efficiency of shape recovery of about 94%. In any case, the shape memory effect alone is unable to heal the damages of pristine PU foam (Figure 8) but can help in the recovery phase to reach the initial spatial configuration (Figure 10). As a result, the PU foam sample with 0.5 wt% PUF/DCPD content allows the balance between shape memory effect and self-healing performance. Therefore, the self-healing property is the result of a compromise between the quantity of microcapsules inserted, and the active quantity after the embedding phase. Moreover, the healing effect comprises two parts: spatial recovery of the walls of the deformed cells caused by the shape-memory effect of the PU foam matrix, and healing of damaged cells by molten PU. The heated PU foam matrix will release the internal stress generated from the compression and bring the walls of damaged cells closer, which is a necessary step to allow self-healing.

The good performances of the PU foam with microcapsules alone assists when associated in the sandwich structure with the shape memory properties of SMPC skins. In fact, as evidenced by the results shown above, the shape memory properties of the sandwich system with SMPC skins and a PU foam core contribute to restoring the initial shape, as visible in the images in Figure 12b. However, without the presence of microcapsules in the foam core, it would not be possible to achieve the healing effect. As a result, the SM composite sandwich only achieved an average healing efficiency of no greater than 65%, largely related to the contribution of the shape-memory effect. In contrast, the presence of only 0.5 wt% of microcapsules in the PU foam core allows it to achieve a healing efficiency up to 98%. Consequently, although the mechanical mixing method and the type of microcapsules used is not currently optimized, an important healing effect can be emphasized by the addition of the same into a polymeric foam during the foaming phase. Then, the ability to optimize and exploit these properties in a sandwich panel with SM properties is very interesting and exploitable in many areas. The possibility of activating the shape recovery and healing effect with a single thermal stimulus following a partial rupture of the structure can be very important in different fields. In fact, this simple process makes this SH-SMPC sandwich structure ideal for a variety of applications, such as aerospace or automotive fields.

## 5. Conclusions

Self-healing systems can be manufactured with a selection of different methods, parameters, and materials. In particular, the combination of shape memory materials and healing systems is one of the paths that is definitely yielding the most advances. For the first time, self-healing and self-repairing of composite structures have been achieved contemporarily. Typical self-healing solutions freeze the damaged structures in the deformed shape after failure. Instead, in this case, heat is provided to recover the initial nominal shape of the damaged composite together with its fixing. Specifically, SM sandwich panels with self-healing properties were fabricated: PUF/DCPD microcapsules were manufactured and then added to a rigid thermosetting polyurethane foam used as a core of a sandwich panel with SMPC skins. Results show that a significant healing effect (up to 89%) is obtained at low filling contents (0.5 wt%) of the healing agents, thus minimally affecting structure performances. To reach this goal, self-healing capsules have been added in the polyurethane foams in the absence of any catalyzer to avoid its detrimental interaction with the liquid resin before and after foaming. Moreover, the polyurethane foam itself showed a shape-memory effect if subjected to heat treatment. The combination of this effect to the SM properties of the SMPC skins within the sandwich structure allows it to achieve a healing efficiency up to 98% with a shape recovery of 98.5% of the system. The spatial proximity due to shape memory effect of PU foam core is at the base of the self-healing process, and the uncured PUF from broken microcapsules can flow to the damages and heal them. Finally, the SM capability of the SMPC skins enable it to recover its initial shape and to lead and partially confine the SM core. The correct combination of material composition and manufacturing procedures has led to the first evidence that the additional functions of shape memory, self-repairing, and self-healing may coexist in future smart composite structures. The ability to design and optimize self-healing shape memory structures at a macroscopic scale rather than synthesize them at the molecular or micrometric level makes these systems suitable for large-scale manufacturing in different fields of application, such as in aeronautics or automotives.

## Figures and Tables

**Table 1:** Physical properties of ESPAK90 components.

Property	Component A	Component B
Viscosity at 25 °C (mPa.s)	1030 ± 100	200 ± 30
Density at 25 °C (kg/L)	1.07 ± 0.02	1.23 ± 0.02

**Table 2:** Measures of manufactured PU foams with and without microcapsules.

Sample	Diameter (mm)	Height (mm)	Weight (g)	Density (g/cm^3^)
PU foam 0 wt%	36.53 ± 0.05	27.71 ± 0.15	2.17	0.12
PU foam 0.5 wt%	36.61 ± 0.14	27.92 ± 0.12	2.04	0.11
PU foam 1 wt%	36.037 ± 0.319	28.86 ± 0.084	2.68	0.12
PU foam 2 wt%	35.953 ± 0.200	30.38 ± 0.12	2.44	0.12
